# Bilateral Morning Glory Syndrome with Bilateral Persistent Fetal Vasculature in a Patient with Joubert's Syndrome

**DOI:** 10.1155/2020/5872758

**Published:** 2020-08-11

**Authors:** Ahmad Bilal Araissi, Alaa Fayed, Youssef Helmy

**Affiliations:** Department of Ophthalmology, Kasr Al-Ainy Hospitals, Cairo University, Giza, Egypt

## Abstract

*Purpose*. We report a case of a 1-year-old girl who was referred to us with a cerebellar anomaly and delayed growth and development for bilateral ptosis and poor fixation. Based on our ophthalmologic examination, we concluded that she has bilateral persistent fetal vasculature (PFV) with morning glory syndrome (MGS). A closer look into her neurologic condition revealed that she has Joubert's syndrome. *Observations*. External examination revealed bilateral symmetrical ptosis with syndromic facies and her fundus examination revealed a large dysplastic optic disc with anomalous radiating vessels and a fibrous tissue tuft originating from the disc. The left eye showed similar findings in addition to a central excavation and a fibrovascular stalk extending from the optic disc. These findings were consistent with bilateral MGS and bilateral PFV. The brain imaging included a computed tomography scan and magnetic resonance imaging, both of which revealed a “molar tooth appearance” of the midbrain and an anomalous cerebellum suggestive of Joubert's syndrome. *Conclusions and Importance*. This is the first case report of a case of bilateral MGS and bilateral PFV associated with Joubert's syndrome. This case report documents the associated optic nerve disease in these patients, not previously described, which are additive causes of visual compromise in addition to the brain insult.

## 1. Introduction

First described in 1969, Joubert's syndrome is a rare group of genetic diseases characterized by congenital malformation of the brainstem and cerebellum. A genetic mutation leads to a defective protein in the structure of primary cilia which is a microtubule-based cellular organelle. Primary cilia are present in nearly all cells; in the eye, a specialized form is present in the outer segment of retinal photoreceptors [1].

The diagnosis is based on a suggestive clinical picture and brain imaging. The distinctive facial features are high rounded eyebrows, broad nasal bridge, ptosis, and low set ears [2]. The universal features of the disease are hypotony, ataxia, and global developmental delay. The pathognomonic feature in brain magnetic resonance imaging is the presence of the “molar tooth sign” which is when elongated superior peduncles (due to the lack of normal decussation) give a radiologic appearance that resembles a molar tooth [3].

Based on the clinical picture, the disease is classified into 8 different subtypes: pure, renal, ocular, oculorenal, orofaciodigital, hepatic, acrocallosal, and the subtype associated with skeletal dysplasia (short ribs, small thorax, short limbs, and renal cystic disease), also called Mainzer-Saldino syndrome [1]. Many other organs can be affected depending on the subtype which includes hepatic fibrosis, oral hamartomas, microcephaly, duodenal atresia, and endocrine disorders [2].

Ocular affection is commonly associated with Joubert's syndrome and ranges from a mild motility defect to severe retinal degeneration. The main ocular manifestations are nystagmus, oculomotor apraxia [4], strabismus, ptosis, coloboma, retinal dystrophy, abnormal retinal pigmentation, retinitis pigmentosa, and optic atrophy [1].

Persistent fetal vasculature (PFV), previously known as persistent hyperplastic primary vitreous, is a congenital persistence of the tunica vasculosa lentis and/or hyaloid system that supplies the fetal lens prior to the production of aqueous. The disease is a spectrum of disorders depending on whether it is anterior (lens) and/or posterior (hyaloid system). It may have a high impact on visual potential depending on whether it is purely anterior, having a better prognosis, or associated with a posterior element where the prognosis is worse. It may also only manifest in the form of a remnant of the hyaloid system (Mittendorf dot or Bergmeister papilla) with no impact on vision [5].

Morning glory syndrome (MGS) is a congenital malformation characterized by an excavated malformed optic disc with a central glial tuft with radiating blood vessels arising from the disc that mostly occurs sporadically and is usually unilateral [6].

## 2. Patient Information

We report the condition of a 1-year-old female infant who was referred to our clinic for bilateral ptosis and poor fixation. The referring neurologist reported delayed growth and development. Their referral posed the possibility of Dandy–Walker syndrome based on a cerebellar anomaly seen in a previous computerized tomography (CT) scan that was not attached. Her history revealed that she was born full-term with no history of neonatal intensive care admission. There was a history of positive consanguinity, but her two older siblings were asymptomatic and there were no similar conditions in the extended family.

## 3. Clinical Findings

**Figure 1:**
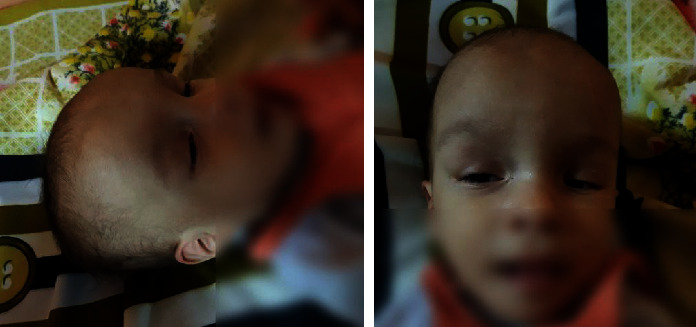
Photograph of the patient. The photo shows the syndromic features including high rounded eyebrows, a broad nasal bridge, and low set ears with bilateral symmetrical ptosis.

The patient had distinct facial features with high rounded eyebrows, a broad nasal bridge, low set ears, and bilateral symmetrical ptosis (Figure 1). The marginal reflex distance (MRD) was 2 mm bilaterally without an apparent lid crease. Levator function could not be assessed. The patient did not appear to fix and follow but did appear to blink to bright light. Both pupils were round regular and sluggishly reactive to light. Anterior segment examination showed bilateral clear corneas with a horizontal diameter of 10 mm, bilateral clear lenses, and intraocular pressure (IOP) of 9 mmHg measured by Perkin's tonometer. Despite the young age of the patient and difficulty to assess the presence of an ocular motility defect, there was no obvious ocular misalignment and the doll's eye movement did not reveal any abnormality.

**Figure 2:**
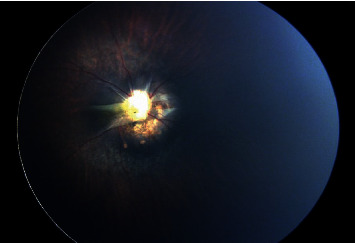
Fundus photography of the right eye. The photo shows mostly attenuated vessels, a small fibrous tuft of tissue originating from the optic disc, and a tessellated midperipheral retina.

Fundus examination of the right eye revealed clear media, attenuated retinal vessels, and a tessellated midperiphery. The right optic disc appeared large-sized and dysplastic with peripapillary atrophy in addition to anomalous radiating vessels that arise from it with peripapillary pigmentation. A fibrous tissue tuft was also noted to be originating from the disc (Figure 2).

**Figure 3:**
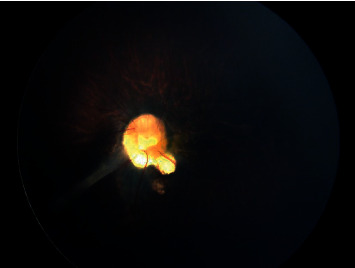
Fundus photography of the left eye. The photo shows attenuated vessels and a fibrovascular stalk extending from the optic disc which was large and dysplastic.

Similarly, the fundus examination of the left eye also showed clear media and attenuated retinal vessels. The left optic disc was larger and dysplastic with a similar anomalous radiating peripapillary vascular appearance, central excavation, and a fibrovascular stalk extending from the optic disc to the nasal retinal periphery (Figure 3). The presence of an optic nerve coloboma was excluded because of the presence of the glial tuft and absence of excavation of any part of the optic nerve, choroid, or retina.

Some pigmentary changes were noted in the retina; however, it does not show signs of any specific type of pigmentary retinopathy as bony spicules and performance of an ERG is difficult due to the young age of the patient and refusal of anesthesiologist to administer anesthesia due to her brain condition.

Based on the clinical picture, the diagnosis of bilateral morning glory syndrome (MGS) and bilateral persistent fetal vasculature (PFV) was made [6].

**Figure 4:**
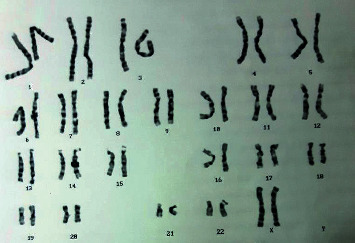
Karyotype study. It shows a normal female karyotype.

**Figure 5:**
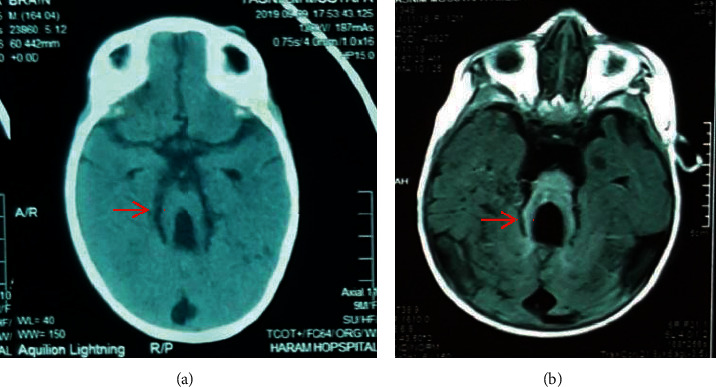
Axial brain imaging. Brain CT scan (a) and brain MRI (b) showing a molar tooth sign at the level of the midbrain (red arrows).

Blood work, including a complete blood count, liver enzymes, and kidney functions were all within normal. An echocardiography to detect any cardiac abnormalities did not reveal any characteristic findings. Karyotyping revealed 46 somatic chromosomes and an XX sex chromosome (Figure 4). New brain imaging was ordered, in the form of a brain computed tomography (CT) scan and magnetic resonance imaging (MRI), both of which revealed a characteristic molar tooth appearance of the midbrain and a small dysplastic vermis (Figure 5). These findings challenged the presumptive diagnosis of Dandy–Walker syndrome and suggested a rare autosomal recessive disease of the cerebellum known as Joubert's syndrome.

**Figure 6:**
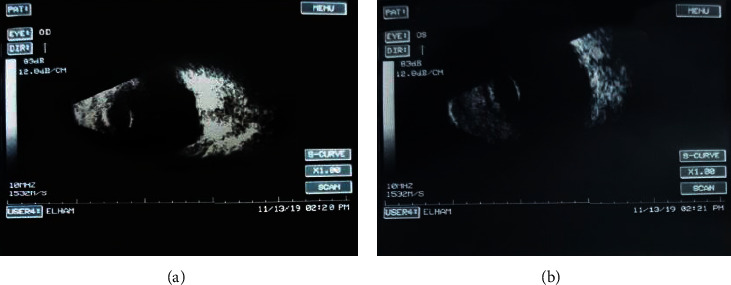
Ocular B-scan ultrasound of the right (a) and left (b) eyes. The ultrasound revealed an axial length of 17 mm bilaterally with no other remarkable findings.

B-scan ultrasonography revealed an axial length of 17 mm bilaterally with no other remarkable findings (Figure 6). Flash visual evoked potential (VEP) was done according to the International Society for Clinical Electrophysiology of Vision (ISCEV) standards, using a flashlight of 8 Hz, and showed no consistent or reproducible responses.

Our findings are suggestive of bilateral morning glory syndrome with bilateral persistent fetal vasculature in a patient with Joubert's syndrome.

## 4. Discussion

We present a rare case of bilateral persistent fetal vasculature and morning glory syndrome in a patient discovered to have Joubert's syndrome. Ocular features of Joubert's syndrome include nystagmus, oculomotor abnormalities, strabismus, and retinal dystrophy. Ptosis has been reported in a minority of patients (29%) and can be unilateral or bilateral with varying severity [1].

Janecke et al. reported 3 cases of a consanguineous Austrian family that had 2 living children and an aborted fetus with Joubert's syndrome and associated MGS [7]. Both children had bilateral MGS and the fetus also showed bilateral MGS on necropsy.

PFV is a predominantly unilateral disease; however, bilateral cases exist in approximately 11% [5]. Most cases have a negative family although Galal et al. reported bilateral PFV in an Egyptian family having a rare autosomal dominant inheritance pattern [8]. Patients with bilateral PFV are reported to be associated with systemic malformations including cleft lip and palate, polydactyly, microcephaly, and early death [5]. Boniuk et al., in a review of PFV, reported that bilateral cases differ in that a majority of cases were microphthalmic (61%) and had a higher incidence of microcornea, glaucoma, retinal detachment, and retinal dystrophies. Kumar et al. reported similar results in addition to a higher incidence of cataract. They also reported that the PFV in these cases is both anterior and posterior [9].

The association of MGS and PFV has been reported in several cases, and the largest series that described this association was by Fei et al. In this case series, 25.88% of patients with MGS were found to have an associated PFV. They reported that the rate of complications was higher and more severe in this group of patients in comparison with isolated cases, including cataract, glaucoma, retinal detachment, strabismus, and nystagmus [6]. The association between MGS and PFV has been postulated to have a genetic etiology. It has been suggested that a PAX6 gene mutation could be the cause since it is involved in several ocular tissue development and was identified in patients with PFV as well as patients with MGS [10].

According to the literature, cases that have combined PFV and MGS are associated with neurologic and facial anomalies [6]; however, to our knowledge, it has not been reported to be present in Joubert's syndrome per say.

Ptosis has been reported in a minority of cases as mentioned earlier; however, the combination of PFV and MGS has not been reported also.

## 5. Conclusions

This is the first case report of a case of MGS and PFV associated with Joubert's syndrome. In contrast to the literature, despite having bilateral PFV, our patient did not have any associated ocular malformations, except for microphthalmia, nor did she have an anterior component of PFV.

This case report documents the associated optic nerve disease in these patients in addition to the already known oculomotor and retinal associations. These optic nerve anomalies are additive causes of visual compromise in addition to the brain insult in this group of patients. Several studies have reported the association of MGS and PFV raising the possibility of a genetic link [6, 8]. The Pax 6 gene is a possible candidate as it is expressed in the nervous system in addition to ocular tissues. Further studies should be carried out to assess the link with Joubert's syndrome.
